# Immediate response mechanisms of Gram-negative solvent-tolerant bacteria to cope with environmental stress: *cis-trans* isomerization of unsaturated fatty acids and outer membrane vesicle secretion

**DOI:** 10.1007/s00253-018-8832-9

**Published:** 2018-02-15

**Authors:** Christian Eberlein, Thomas Baumgarten, Stephan Starke, Hermann J. Heipieper

**Affiliations:** 1 Department of Environmental Biotechnology, Helmholtz Centre for Environmental Research—UFZ, Permoserstr. 15, 04318 Leipzig, Germany; 2 Center for Biomembrane Research, Department of Biochemistry and Biophysics, Stockholm University, Svante Arrhenius väg 16C, 10691 Stockholm, Sweden

**Keywords:** Biofilm formation, Gram-negative cell wall, Surface hydrophobicity, Lipopolysaccharide layer, Membrane micro domains, Phospholipids

## Abstract

Bacteria have evolved an array of adaptive mechanisms enabling them to survive and grow in the presence of different environmental stresses. These mechanisms include either modifications of the membrane or changes in the overall energy status, cell morphology, and cell surface properties. Long-term adaptations are dependent on transcriptional regulation, the induction of anabolic pathways, and cell growth. However, to survive sudden environmental changes, bacterial short-term responses are essential to keep the cells alive after the occurrence of an environmental stress factor such as heat shock or the presence of toxic organic solvents. Thus far, two main short-term responses are known. On the one hand, a fast isomerization of *cis* into *trans* unsaturated fatty leads to a quick rigidification of the cell membrane, a mechanism known in some genera of Gram-negative bacteria. On the other hand, a fast, effective, and ubiquitously present countermeasure is the release of outer membrane vesicles (OMVs) from the cell surface leading to a rapid increase in cell surface hydrophobicity and finally to the formation of cell aggregates and biofilms. These immediate response mechanisms just allow the bacteria to stay physiologically active and to employ long-term responses to assure viability upon changing environmental conditions. Here, we provide insight into the two aforementioned rapid adaptive mechanisms affecting ultimately the cell envelope of Gram-negative bacteria.

## Introduction

The envelope of Gram-negative bacteria is the outermost barrier of the cell, and it consists of an outer membrane with lipopolysaccharides (LPS) facing the extracellular space and an inner membrane enclosing the cytoplasm. The space between these membranes, known as periplasm, contains a peptidoglycan layer. While the outer membrane is not permeable throughout most of its surface, non-specific porins allow the passive diffusion of molecules (Hancock et al. 1979; Nakae 1976; van den Berg 2012). The inner membrane represents the main diffusion barrier of the Gram-negative cell, and it is very susceptible to stressors like hydrocarbons or elevated temperatures. Both stressors increase directly the membrane fluidity, either due to the accumulation of hydrophobic substances in the membrane or due to an increased mobility of phospholipids (Beney and Gervais 2001; Hazel and Williams 1990; Heipieper and de Bont 1994). This can lead to the disruption of essential functions of the membrane as enzymatic matrix and diffusion barrier resulting in the collapse of electrochemical gradients (Isken and de Bont 1998). To maintain a certain degree of membrane fluidity, the membrane composition can be altered, a process known as homeoviscous adaptation (Sinensky 1974). To counteract increasing membrane fluidity, the synthesis of saturated phospholipid fatty acids is a common mechanism in Gram-negative bacteria (Ingram 1977; Kabelitz et al. 2003; Suutari and Laakso 1994). Phospholipids containing saturated fatty acids display a much higher transition temperature than those with unsaturated fatty acids, thereby decreasing membrane fluidity. For example the melting point of phospholipids with C16:0 is about 63 °C higher than that of phospholipids with 16:1 *cis*-unsaturated fatty acids (Roach et al. 2004; Zhang and Rock 2008). However, in a situation in which the bacterial cell is facing severe and fast environmental changes (e.g. high concentrations of toxic substances) resulting in elevated mobility of membrane components, the comparatively slow-acting de novo synthesis of saturated fatty acids cannot keep up. Moreover, the de novo synthesis of saturated fatty acids is an energy-consuming process which cannot be employed in conditions where cell viability is impaired (Heipieper et al. 2007). Consequently, the survival of the cell depends on adaption mechanisms acting immediately and which are independent from transcriptional regulation and growth. Notably, these mechanisms ensure the survival until growth-dependent mechanisms complete the adaptive response (Cronan 2002; Hartig et al. 2005; Heipieper et al. 2007; Zhang and Rock 2008). As the cell envelope of Gram-negative bacteria is affected first by emerging environmental stress, both fast adaptive responses described in the following sections involve changes of the interface between the cell and its surrounding. It is important to notice that the inner as well as the outer membrane can be altered by mechanisms taking effect fast but differ fundamentally in their mode of action. Which of the two membranes could be modified by which mechanisms and how will be explained and discussed below.

## *Cis-trans*-isomerization of membrane fatty acids

The cytoplasmic membrane of most Gram-negative bacteria consists of saturated and monounsaturated fatty acids. For a long time, it was believed that unsaturated membrane fatty acids always exhibit the *cis* configuration, until in the mid-1980s evidence for the existence of *trans* unsaturated fatty acids in *Vibrio* and *Pseudomonas* was presented (Guckert et al. 1986; Guckert et al. 1987). Ultimately, it was discovered that the conversion of *cis* unsaturated fatty acids into their corresponding *trans* configuration represents a strategy to withstand elevated temperatures in *Vibrio* sp. strain ABE-1 and toxic hydrocarbons in *Pseudomonas putida* P8 (Heipieper et al. 1992; Okuyama et al. 1991). In both organisms, the amount of *trans* fatty acids was increased by simultaneous decrease of their *cis* form. In *P. putida* P8, it could be shown that the amount of *trans* fatty acids correlates positively with the concentration of the stressor phenol added to the cultures and even non-dividing cells showed the conversion of *cis* to *trans* (Heipieper et al. 1992)*.* Further, the *cis*-*trans* conversion is not affected by inhibition of the fatty acid biosynthesis with cerulenin and is independent from the de novo synthesis of proteins (Heipieper et al. 1992; Kiran et al. 2005). Therefore, the *cis*-*trans* conversion is a powerful tool during growth-inhibiting conditions where the general fatty acid composition cannot be changed (Morita et al. 1993). The applicability as a fast adaptive response is conferred by the fact that it works constitutively and that highest amounts of the *trans* fatty acids are accumulated already 30 min after the initiated stress. Moreover, neither ATP nor cofactors are required to perform the *cis*-*trans* conversion (von Wallbrunn et al. 2003). Furthermore, *cis*-*trans* conversion is not affected by chloramphenicol, which blocks protein translation. Hence, the *cis*-*trans* isomerization is independent from the de novo protein biosynthesis (Heipieper et al. 1992; Kiran et al. 2005).

The conversion from *cis* to *trans* fatty acids is carried out by the periplasmic protein Cti, the *cis*-*trans-*isomerase. Predominant substrates for the enzyme are the fatty acid residues palmitoleic acid (C16:1Δ9*cis*) and *cis*-vaccenic acid (C18:1Δ11*cis*) of phospholipids (Heipieper et al. 1992; Heipieper et al. 2003). The position of the double bond remains the same after the reaction. This was shown when growth media of *P. putida* was supplemented with oleic acid (C18:1Δ9*cis*). This fatty acid is not naturally produced by *P. putida* but incorporated into the phospholipids of the membrane. After exposure to solvent stress with 4-chlorophenol, the direct conversion to the corresponding *trans* isomer elaidic acid (C18:1Δ9*trans*) took place and oleic acid was depleted (Diefenbach and Keweloh 1994). Further proof for that the double bond remains at the same position during the isomerization was provided by the fact that the sum of the amount of both isomers kept constant regardless of the toxin concentration (Heipieper et al. 1992). But how exactly contributes an increased *trans/cis* ratio to a more rigid membrane? Under non-stressed conditions, unsaturated fatty acids exhibit the *cis* configuration, and the double bond present displays an unmovable bend of 30° (Macdonald et al. 1985; Roach et al. 2004). This kink leads to steric hindrance within the fatty acid residues enhancing membrane fluidity. In contrast, in *trans* unsaturated fatty acids, the kink adds up to only 6° (Macdonald et al. 1985). Thus, compared to the *cis* isomers, *trans* fatty acids can align more closely to each other and in this regard resemble saturated fatty acid residues (Heipieper et al. 2003; Kulig et al. 2016; Pierce et al. 2014). Consequently, this results in a more rigid membrane (Chen et al. 1995; Diefenbach et al. 1992). This was also demonstrated by comparing the behavior of phospholipids containing *trans* fatty acids with those containing the corresponding *cis* fatty acids in model membranes and by molecular dynamics (Roach et al. 2004).

*Cis-trans*-isomerization can be induced by the accumulation of different hydrocarbons and phenolic compounds in the cytoplasmic membrane. Their ability to accumulate in the membrane depends on their hydrophobicity given as log P_ow_ value. This value is the partitioning coefficient of a compound between octanol and water (Laane et al. 1987). Organic hydrocarbons with a P_ow_ value between 1 and 4 preferentially partition inside the membrane and thus are highly toxic for microorganisms (Heipieper and de Bont 1994; Sikkema et al. 1995; Weber and de Bont 1996). Notably, the degree of *cis* to *trans* conversion correlates with the toxicity/hydrophobicity and the concentration of toxic organic hydrocarbons but does not depend on a distinctive chemical structure of the stressor (Heipieper et al. 1995; Neumann et al. 2005). Besides various toxic organic compounds, osmotic stress, the presence of heavy metals, and antibiotics acting on the membrane result in an increase of the *trans*/*cis* ratio (Hachicho et al. 2014; Heipieper et al. 1996; Isken et al. 1997; Kotchaplai et al. 2017; Piotrowska et al. 2016). This reflects the importance of the system as general stress response mechanism in Gram-negative bacteria (Ramos et al. 2001; Segura et al. 1999; Zhang and Rock 2008). In contrast to that, Cti cannot be employed to adjust the membrane fluidity if temperatures drop because the conversion from *trans* to *cis* is not catalyzed but requires the de novo synthesis of unsaturated fatty acids, and the amount of *trans* fatty acids is low in Gram-negative bacteria. It has to be mentioned that the effectiveness of *cis-trans*-isomerization is not as high as the replacement of unsaturated fatty acids by saturated ones (Macdonald et al. 1985; Roach et al. 2004). But, especially if a fast reaction to environmental stress is needed, the conversion of *cis* fatty acids contributes substantially to the survival of the cell and allows subsequent long-term adaptations to take place. This has been demonstrated for heat shock conditions and the exposure of cells to toxic hydrocarbons (Heipieper et al. 2003; Kiran et al. 2005; Weber and de Bont 1996).

So far, *cis-trans*-isomerization was shown to be employed as adaptive response to stress in strains of all known *Pseudomonas sp.* (Heipieper et al. 1992; Molina-Santiago et al. 2017), *Vibrio* sp*.* (Okuyama et al. 1991), *Methylococcus capsulatus* (Löffler et al. 2010), *Alcanivorax borkumensis* (Naether et al. 2013), and *Colwellia psychrerythraea* (Hashimoto et al. 2015). Table 1 summarizes the results of a recent BLASTP analysis using the predicted amino acid sequence from the *cti* gene of *P. aeruginosa* PAO1 as reference and reveals that the system might also be present in other microorganisms. Although physiological evidence is not yet provided, a promising candidate for bearing *cis-trans*-isomerization activity is *Nitrosomonas* since the genera contains *trans* fatty acid residues in its phospholipids (Keweloh and Heipieper 1996). By performing alignments of the peptide sequence, it was found that almost all Cti proteins bear an *N*-terminal signal sequence (except the one from *Vibrio cholera*) suggesting a periplasmic location of the enzyme; this was confirmed for *P. oleovorans* and *P. putida* DOT-T1E (Junker and Ramos 1999; Pedrotta and Witholt 1999). Phylogenetic analysis revealed that Cti proteins from the genera *Pseudomonas* and *Vibrio* diversified into three branches from a common ancestor. The largest group is composed out of proteins from *P. putida*, *P. aeruginosa*, *Pseudomonas sp.*, and *V. cholerae* while the latter clearly emanates from the others (von Wallbrunn et al. 2003). Although the effective and simple c*is-trans*-isomerization is not a common feature present in Gram-negative bacteria, it presumably contributes to the high adaptability of particularly *Pseudomonas* and *Vibrio* species conquering diverse ecosystems and showing a high durability in environmental adverse situations. This idea is supported by the increased tolerance towards octanoic acid of a genetically modified *Escherichia coli* strain containing the Cti from *P. aeruginosa* (Tan et al. 2016). It is noteworthy that the expression of *cti* improved the robustness of the *E. coli* strain and concomitantly facilitates biotechnological downstream processing. For biochemical characterization, the Cti protein from *P. putida* P8 was recombinantly expressed in *E. coli* (Holtwick et al. 1997) or purified from periplasmic fractions of *P. oleovorans* and *Pseudomonas* sp. E-3 (Okuyama et al. 1998; Pedrotta and Witholt 1999). Cti is a neutral 87 kDa protein, monocistronically transcribed and continuously expressed (Kiran et al. 2005). After translocating the protein to the periplasm, the *N*-terminal hydrophobic signal sequence is cleaved off (Holtwick et al. 1997; Junker and Ramos 1999; Pedrotta and Witholt 1999). Cti is a cytochrome *c*-type protein as it is bearing a heme-binding motif (Holtwick et al. 1999). For *P. putida*, it was proposed that iron (probably Fe^3+^) is essential for the reaction catalyzed (Okuyama et al. 1998). This is in agreement with observations of strongly diminished Cti activity after site-directed mutagenesis of the heme-binding site as well as shutting down the assembly machinery of heme groups to cytochrome *c*-type proteins in *P. putida* P8 (Holtwick et al. 1999). Via the putative Fe^3+^ coordinated by the heme-binding site, an enzyme substrate complex is formed and an electron is temporarily removed from the *cis*-double bond through electrophilic attack changing the sp^2^ hybridization to sp^3^, thus enabling the free rotation of the C–C bond. After establishing the *trans* configuration, the electron is transferred back reforming the double bond (Okuyama et al. 1998; von Wallbrunn et al. 2003). Other cofactors like NAD(P)H, glutathione, or ATP are not necessary to carry out the reaction (Diefenbach et al. 1992; Heipieper and de Bont 1994; Heipieper et al. 1992).

**Table 1:** Protein BLAST-analysis (basic local alignment search tool) with *cis-trans*-isomerase sequence of *Pseudomonas aeruginosa* PAO1 as a reference (National Center for Biotechnology, NCBI)

Organism	Query coverage	Identity	Accession number
*Pseudomonas aeruginosa*	100%	100%	WP_003113604.1
*Pseudomonas putida*	100%	99%	KTK92302.1
*Acinetobacter baumannii*	100%	99%	SCY60049.1
*Pseudomonas denitrificans*	98%	99%	WP_049316913.1
*Pseudomonas citronellolis*	98%	79%	WP_061561289.1
*Pseudomonas delhiensis*	98%	78%	WP_089390327.1
*Pseudomonas knackmussii*	100%	76%	WP_043253302.1
*Pseudomonas nitroreducens*	99%	76%	WP_084359066.1
*Pseudomonas alcaligenes*	97%	74%	WP_021216801.1
*Pseudomonas fluorescens*	99%	69%	WP_011334494.1
*Streptococcus pneumoniae*	98%	66%	CJK83205.1
*Stenotrophomonas rhizophila*	100%	65%	KWW19403.1
*Alcanivorax dieselolei*	99%	56%	WP_014993834.1
*Alcanivorax xenomutans*	96%	56%	WP_080530670.1
*Zobellella denitrificans*	96%	53%	ATG75919.1
*Nitrosomonas ureae*	96%	52%	SEF93585.1
*Methylophaga aminisulfidivorans*	96%	51%	WP_007146066.1
*Marinobacter antarcticus*	98%	51%	WP_072795224.1
*Oceanospirillales bacterium*	98%	51%	PCI79966.1
*Polycyclovorans algicola*	96%	51%	WP_029889450.1
*Nitrosomonas europaea*	97%	50%	KXK36297.1
*Solimonas flava*	99%	50%	WP_084615840.1
*Nevskia ramosa*	96%	49%	WP_051144340.1
*Cycloclasticus sp. P1*	96%	45%	WP_041230499.1
*Methylobacter luteus*	96%	43%	WP_027158246.1
*Labrenzia alba*	98%	42%	CTQ60836.1
*Methylococcus capsulatus*	97%	41%	WP_017364148.1
*Methyloterricola oryzae*	98%	40%	WP_052808280.1
*Desulfoluna spongiiphila*	96%	40%	WP_092211474.1
*Methylosarcina lacus*	96%	40%	WP_024297537.1
*Variovorax sp. URHB0020*	96%	40%	WP_028253212.1
*Methylomonas methanica*	97%	39%	WP_013818496.1
*Vibrio litoralis*	99%	39%	WP_027695248.1
*Aliivibrio wodanis*	96%	39%	WP_061012074.1
*Bdellovibrio bacteriovorus*	99%	38%	WP_081111064.1
*Nitrosospira multiformis*	97%	38%	WP_081346773.1
*Nitrosospira sp. Nsp13*	97%	38%	WP_090907238.1
*Colwellia sp. PAMC 21821*	96%	38%	WP_081180022.1
*Alteromonas macleodii*	96%	38%	WP_014976105.1
*Vibrio algivorus*	99%	38%	WP_089124128.1
*Pseudoalteromonas rubra*	96%	38%	WP_058796649.1
*Vibrio mytili*	96%	37%	WP_041153942.1
*Vibrio antiquarius*	96%	37%	WP_081442549.1

**Fig. 1:**
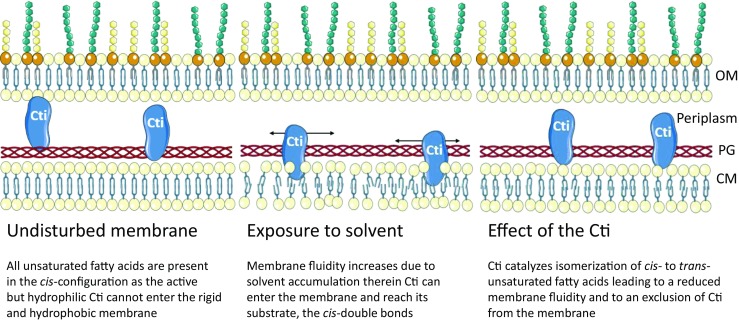
Possible regulation of periplasmic *cis-trans*-isomerase (Cti) by membrane fluidity in case of exposure to environmental stress. OM outer membrane, CM cytoplasmic membrane, PG peptidoglycan layer (modified after Heipieper et al. 2003; Heipieper et al. 1996)

Characteristically for immediate response mechanisms, no transcriptional regulation exists for Cti (Kiran et al. 2005). Instead, the activity of the constitutively expressed enzyme has to be regulated differently. Cells deprived in energy sources and also non-dividing cells show *cis-trans*-isomerization; hence, a complex model for regulation involving enzymatic pathways can presumably be ruled out (Heipieper et al. 1992). The regulation is probably simply done by enabling the access of the enzyme to its substrate, which can be controlled via membrane fluidity (Fig. 1). Under non-stressed conditions, the membrane is relatively rigid and *cis* fatty acid residues of phospholipids are not accessible for the hydrophilic Cti since they are embedded in a certain depth of the membrane. The presence of hydrocarbons partitioning in the membrane or high temperature increases the membrane’s fluidity substantially (Hartig et al. 2005; Heipieper et al. 2001). In this process, gaps in a more disordered membrane open up allowing Cti to bind to and react with its substrate. As more *cis* fatty acid residues are converted to *trans*, acyl chains are packed tighter, thereby pushing Cti out of the membrane (Chen et al. 1995; Loffhagen et al. 2007; Roach et al. 2004; Seelig and Waespe-Sarcevic 1978). In other words, a certain fluidity of the membrane is necessary for Cti to reach its substrate inside the membrane and reduction of membrane fluidity due to *cis-trans*-isomerization counteracts the intrusion of the isomerase (Heipieper et al. 2003; Heipieper et al. 1996). This model explains the observed correlation between the extent of c*is-trans*-isomerization and the toxicity caused by different concentrations of specific environmental stressors (Heipieper et al. 1995; Heipieper et al. 1996). In addition, the model is in accordance with the fast onset of this regulatory mechanism, and as soon as the membrane fluidity surpasses or falls below a certain threshold, Cti becomes active or inactive respectively (Heipieper et al. 2001).

The application of the *trans*/*cis* ratio and the formation of *trans* unsaturated fatty acids as stress biomarker in environmental samples or during in situ bioremediation processes was discussed, and it was proposed that a *trans*/*cis* ratio higher than 0.1 could indicate the occurrence of environmental stress (Guckert et al. 1986; Guckert et al. 1987; Heipieper et al. 1995; Pinkart et al. 1996). However, it was proven that an increased amount of *trans* unsaturated fatty acids may not be a suitable benchmark to evaluate environmental stress scenarios, especially for long-term contaminated sites (Fischer et al. 2010). Due to its nature as urgent stress response, an increased *trans*/*cis* ratio can be measured directly after a contamination event, but with the onset of long-term adaptations, the *trans*/*cis* ratio decreases to levels of an unstressed reference (Fischer et al. 2010). Moreover, it is not possible to assign whether a changing phospholipid fatty acid pattern is derived from alterations on the membrane or the species level (Frostegard et al. 2011).

## Formation of OMVs

Besides adapting the inner membrane in response to environmental stress, Gram-negative bacteria have evolved defense mechanisms altering the outer membrane. The outer membrane consists of a distal lipopolysaccharide (LPS) layer and a corresponding proximal phospholipid monolayer, which includes linkers to the peptidoglycan layer in the periplasm. Characteristic for the LPS layer are three different components: lipid A, an amphipathic glycolipid; the core oligosaccharide; and the most distal, the O-specific polysaccharide (also known as O-antigen) region. Compared to the inner membrane, adaptive mechanisms affecting the outer membrane are far less understood (de Carvalho et al. 2009; Heipieper et al. 1991; Neumann et al. 2006). However, the formation of outer membrane vesicles (OMVs), which are spherical particles with a diameter between 20 and 500 nm released from the outer membrane into the extracellular space, has been studied in detail (Schwechheimer and Kuehn 2015, Beveridge 1999; Kuehn and Kesty 2005; Mashburn-Warren et al. 2008b). The release of OMVs is a common phenomenon among Gram-negative bacteria (Kulp and Kuehn 2010; Schwechheimer et al. 2013; Zhou et al. 1998), and it is attributed to the pathogenicity of strains like *P. aeruginosa* causing cystic fibrosis (Kadurugamuwa and Beveridge 1997). Moreover, Gram-negative bacteria forming OMVs are present in any environment, and sessile as well as planktonic cells have been found to release OMVs (Beveridge 1999; Beveridge et al. 1997; Biller et al. 2014; Brandtzaeg et al. 1992; Hellman et al. 2000; Hickey et al. 2015). The biogenesis of OMVs and their numerous functions besides stress adaptation have been recently reviewed in great detail (Kulp and Kuehn 2010; Schertzer and Whiteley 2013; Schwechheimer and Kuehn 2015). The function of OMV release as a stress response was shown in several reports. For example, when *P. aeruginosa* was treated with the antibiotic ciprofloxacin or hydrogen peroxide, the bacteria responded with increased vesicle production (Macdonald and Kuehn 2013; Maredia et al. 2012). In addition, the production of OMVs in *P*. *putida* as response to the exposure to solvents, high temperature, and EDTA was shown before (Baumgarten et al. 2012b; Baumgarten et al. 2012a). The reason why the formation of OMV is beneficial during stress conditions will be approached and explained in the following section.

**Fig. 2:**
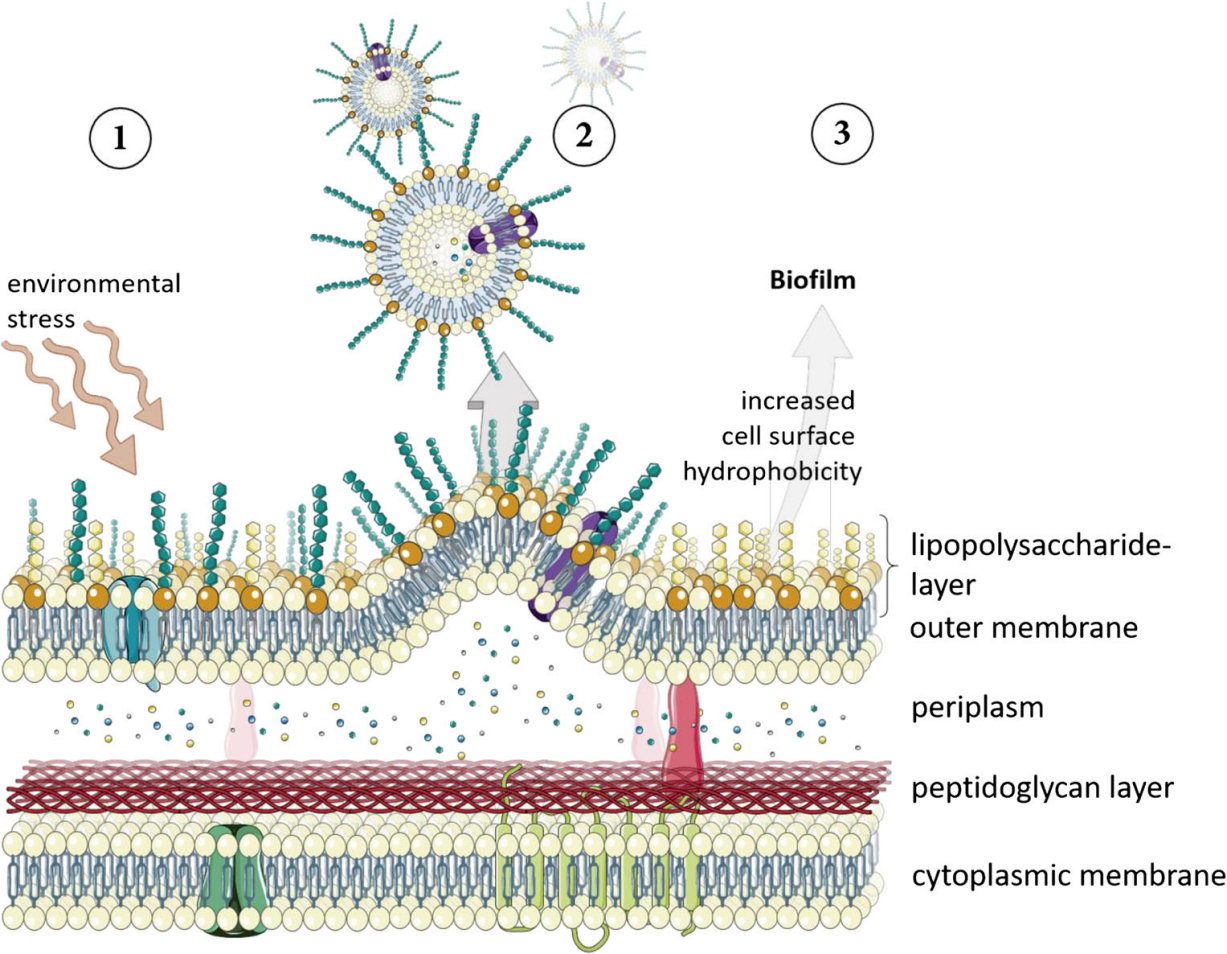
Scheme of Gram-negative cell wall with membrane micro domain associated LPS types. The formation of outer membrane vesicles from a certain membrane micro domain is shown. 1 Environmental stress (e.g., temperature, solvents, EDTA, osmotic stress) on bacterial outer membrane. 2 Subsequent bulging out of the outer membrane and vesicle detachment resulting in a depletion of hydrophilic LPS type (blue). 3 Concomitant enrichment of hydrophobic LPS type (yellow) in the outer membrane leads to enhanced ability to form biofilms

Certain stress responses in Gram-negative bacteria can be found in many genera, potentially being present ubiquitously (Heipieper et al. 2007; Ramos et al. 2001; Segura et al. 1999), and the modification of bacterial surface properties like surface charge and hydrophobicity is one of those (Wick et al. 2003; Wick et al. 2002). The latter can be measured as the contact angle (θ_w_) between a bacterial lawn and a droplet of water (van Loosdrecht et al. 1987). In several bacteria, an increase in the surface hydrophobicity was observed when cells were incubated with solvents or exposed to osmotic stress (Baumgarten et al. 2012a; Hachicho et al. 2017; Löffler et al. 2010; Naether et al. 2013; Neumann et al. 2006; Wick et al. 2002). This enhanced hydrophobicity was observed to be a rapid response (within 10 min) and was also proven to be of physiological origin since dead cells did not show such a reaction. Moreover, after withdrawal of the solvent, the hydrophobicity recovered slowly but steadily to its previous status most probably due to the de novo synthesis of LPS components (Neumann et al. 2006). In *P. aeruginosa*, the O-specific region of the LPS layer contains two major components (Makin and Beveridge 1996). The A-band is a rather hydrophobic and low-molecular-weight LPS, consisting of a homopolymer of d-rhamnose and small amounts of 2-keto-3-deoxyoctonic acid. The second component, the B-band, is a rather hydrophilic and high-molecular-weight LPS, consisting of a heteropolymer of mainly uronic acid derivatives and *N*-acetylfucosamine (Sabra et al. 2003). In *P. aeruginosa*, a depletion of B-band components in the LPS layer was observed after exposure to high temperature, membrane-active antibiotics, or at low oxygen saturation (Makin and Beveridge 1996) and a similar response was reported for *P. putida* after exposure to *o*-xylene (Pinkart et al. 1996). The assumption that the depletion of the B-band is derived from its export within OMVs is supported by several observations. In *P*. *aeruginosa*, formation of OMVs was reported after treatment with hydrogen peroxide and vesiculation was dependent on the ability of the bacterium to form B-band LPS (Macdonald and Kuehn 2013). Moreover, an enrichment of B-band LPS in the constitutively produced OMVs of *P. aeruginosa* was documented earlier (Li et al. 1996). Consistent with that is the report of hypersensitivity towards oxidative stress caused by the deficiency to synthesize B-band LPS in Gram-negative *Enterobacterium* (Berry et al. 2009). In addition, a number of reports state that OMVs formed in cultures of *P. aeruginosa* were enriched with certain LPS components after stress exposure (Berry et al. 2009; Deatherage et al. 2009; Kadurugamuwa and Beveridge 1995; Sabra et al. 2003; Schooling and Beveridge 2006). Taken together, the hydrophilic B-band LPS is preferably exported via OMVs whereas the hydrophobic A-band LPS remains in the bacterial outer membrane. This means that the amount of OMVs produced determines the increase in cell surface hydrophobicity. A conceivable opinion that solvents are repelled with a more hydrophilic surface (de Bont 1998) is obviously invalid, but what is the advantage of a hydrophobic surface? Recently, cultures of *P. putida* stressed with solvents, NaCl, EDTA, or heat were proven to have an enhanced ability to form biofilms after increasing their hydrophobicity (Baumgarten et al. 2012b). Moreover, the OMVs detected in the culture supernatants after application of stress showed proteomic and lipidomic composition typical for the outer membrane. That OMVs could be involved in biofilm formation was suggested before (Beveridge et al. 1997). Biofilms or microcolonies are microbial agglomerations embedded in extracellular polymeric substances (EPSs), attached to surfaces and displaying a different phenotype than planktonic cells (Costerton et al. 1978; Donlan and Costerton 2002). The major steps in biofilm formation are adhesion of planktonic cells to the surface; the initiation and maturation of the agglomeration; and finally, the dispersion of cells from the biofilm (Stoodley et al. 2002). Bacteria growing in biofilms are known to have considerable advantages over the planktonic lifestyle. Bacterial cells are protected within the biofilm; hence, the capability to withstand and tolerate harsh environmental conditions as treatment with antimicrobial compounds, heat, or solvents is increased (Beveridge et al. 1997; Ceri et al. 1999; de Carvalho et al. 2009; Kim et al. 2012; Weitere et al. 2005). The biofilm consists out of cells, EPS, proteins, DNA, and other macromolecules (Sutherland 2001) but also OMVs contribute to the biofilm matrix (Beveridge et al. 1997; Schertzer and Whiteley 2012; Schooling and Beveridge 2006). In addition to the stabilization of the biofilm, its formation is also enhanced a lot due to the fact that cells forming vesicles turn more hydrophobic as discussed above. Subsequently, this favors an attachment of cells to each other and to a hydrophobic surface. To sum up, Fig. 2 shows a general stress response cascade in Gram-negative bacteria that is most likely initiated by vesiculation conferring a higher surface hydrophobicity to the cells that subsequently results in an enhanced ability to form biofilms or microcolonies (Baumgarten et al. 2012b).

Various theories how OMVs are formed are currently discussed in literature (Schwechheimer and Kuehn 2015). In this regard, one of the best investigated strains is *P. aeruginosa* because of its importance as pathogen causing cystic fibrosis. Here, the conversion of 2-heptyl-4-quinolone (HHQ) into 2-heptyl-3-hydroxy-4-quinolone, the so-called *Pseudomonas* quinolone signal (PQS), by the cytoplasmic monooxygenase PqsH and the subsequent export of PQS stimulates the formation of OMVs (Diggle et al. 2006; Florez et al. 2017; Schertzer et al. 2010). PQS has a heteroaromatic structure with a C7 aliphatic residue. Due to its amphiphilic character, the molecule interacts with both the 4′-phosphate and acyl chains of lipid A (Mashburn-Warren et al. 2008a) and thus can accumulate in the LPS layer promoting vesicle blebbing (Schertzer et al. 2009). In addition, in *P. aeruginosa*, a sequestration of the PQS molecule in the inner membrane, which could lead to rapid OMV production once further forwarded to the outer membrane, was discussed (Florez et al. 2017). Vesiculation induced specifically by PQS seems not to be a general feature among Gram-negative bacteria since already among the same genus, the strain *Pseudomonas putida* KT2440 does have neither any homologous genes for the synthesis of PQS nor any other known quorum sensing molecule but seems to regulate population density through a so far unknown signal molecule (Espinosa-Urgel and Ramos 2004; Fernandez-Pinar et al. 2008). Trace amounts of HHQ were detected in *P. putida* culture supernatants but with concentrations at least one order of magnitude lower than in *P. aeruginosa* (Dubern and Diggle 2008). Nevertheless, supported by the fast formation of OMVs in *P. putida*, *P. aeruginosa*, and *E. coli* (Baumgarten et al. 2012a; Makin and Beveridge 1996; Manning and Kuehn 2011; Neumann et al. 2006), the existence of several different signal molecules or other mechanisms seems plausible to secure the ability to act immediately upon emerging environmental stress. Hereby, amphipathic molecules able to penetrate the outer membrane are conceivable candidates. Moreover, the existence of hotspots for vesiculation would explain how such a process is controlled without inducing membrane blebbing all over the bacterial surface with concomitant risk of cell lysis. Consistent with that is a more recent understanding of the membrane displaying a rather heterogeneous than homogeneous pattern. So-called membrane micro domains (also known as lipid rafts) may be formed by an enrichment of certain proteins or glycolipids (Bramkamp and Lopez 2015; Engelman 2005). Membrane micro domains where OMV formation preferably originates could contain an enrichment of B-band LPS. Diminished crosslinks between the outer membrane and the peptidoglycan (Kulp and Kuehn 2010; McBroom et al. 2006; Schwechheimer and Kuehn 2013; Wessel et al. 2013) would further promote the vesiculation process.

Since both adaptive mechanisms act on the Gram-negative cell envelope at first, it may not always be clear which effect can be traced back to which mechanism. However, since the outer membrane is of asymmetric architecture, the number of phospholipids is much smaller than in the inner membrane. Hence, the targets for Cti are less abundant and in turn resulting the cytoplasmic membrane to be the main domain affected by *cis-trans*-isomerization. Nonetheless, the fatty acid composition of OMV preparations of solvent-stressed cells revealed an elevated *trans*/*cis* ratio indicating that the Cti is also acting in part on the outer membrane (Baumgarten et al. 2012b). To elucidate this further would be an interesting research topic and also would tell more about Cti’s scope of action.

In summary, the formation of OMVs is a ubiquitous mechanism in Gram-negative bacteria. Presumably, a basal production of vesicles is always present and vesiculation can be increased in certain scenarios (Schwechheimer and Kuehn 2015). To elucidate the probably diverse mechanisms of vesiculation, its regulation remains a challenging research field. As proposed for *cis-trans*-isomerization (Heipieper et al. 2003; Heipieper et al. 1996) also for OMV formation, a rather simple trigger than a complex regulation is conceivable to assure that the cell can immediately act independent of growth and energy supply. In bacteria bearing both adaptational mechanisms, most probably, they take place at the same time in a stress situation due to their passive activation. This phenomenon, a concomitant increase in surface hydrophobicity as well as for the *trans*/*cis* ratio, was already observed for bacteria in stress situations (Löffler et al. 2010; Naether et al. 2013). However, in contrast to *cis-trans*-isomerization, it was demonstrated that neither non-growing cells nor dead cells could increase their hydrophobicity fast and efficient upon stress exposure or showed an enhanced ability to form biofilms (Baumgarten et al. 2012b; Neumann et al. 2006). Both urgent response systems, the *cis-trans*-isomerization as well as the formation of OMVs, secure a fast alteration and adaptation in the Gram-negative bacteria cell envelope to prevent a deadly impact of emerging adverse environmental conditions. Concomitantly, both responses can provide sufficient time for further more complex but also slower-acting adaptive cascades eventually completing the adaptation process (Cronan 2002; Hartig et al. 2005; Zhang and Rock 2008).
